# Year in review: 2024–2025

**DOI:** 10.1016/j.stemcr.2025.102513

**Published:** 2025-06-10

## Main text

**Figure:**
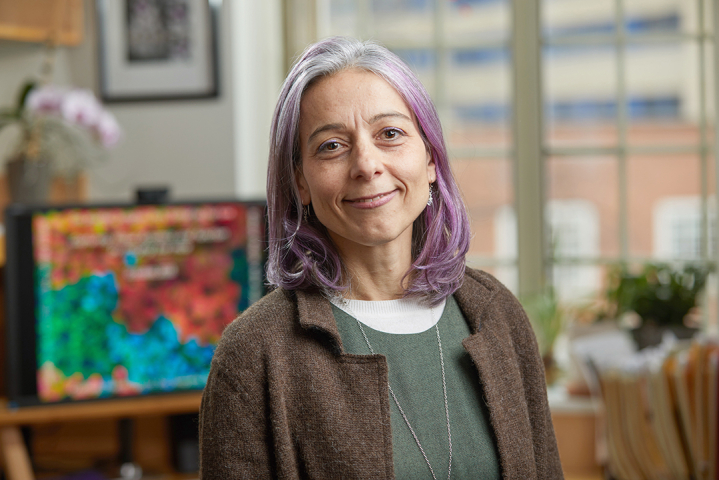
Valentina Greco, ISSCR President 2024–2025

As I reflect on this past year as International Society for Stem Cell Research (ISSCR) President, I feel gratitude for what I have learned and the outlook it has given me on the Society and on the field it represents. I will share a few reflections in the following.

As I began my role, I realized that I had only a superficial understanding of the work of more than 200 volunteers and ISSCR team members. With Director of Media and Strategic Communications Kym Kilbourne, I embarked on monthly interviews and email correspondence with colleagues in leadership roles to learn about the work of the ISSCR committees, task forces, and working groups. It has been humbling to listen to them and to experience the passion and commitment each of them brings to our Society. Their work spans large subfields of regenerative biology and medicine allowing us to form public and regulatory policy priorities, educate ourselves and others, participate in manufacturing innovations, and wrestle with important ethical questions, to name just a few. It was a privilege to report on their work monthly in my president’s messages.

The work of the Society’s 200 volunteers is simply extraordinary. This year, we recognized them all, as a group, with the 2025 ISSCR Public Service Award. I had the privilege to deliver the news to all of them, and two things struck me. First, the deep gratitude that many volunteers articulated in their replies. Whether they were established or earlier in their careers, I felt they experienced the recognition with excitement. It reminded me how much we take for granted the impact that expressing gratitude has on us all. Second, I was in awe to witness, for the first time, ISSCR leadership giving a collective award. It crystalized for me the importance of celebrating groups in addition to highlighting individual achievements. Further, it made me think of the cost when we do not celebrate the many who contribute to everything we do.

I also listened to members to learn how they perceive the ISSCR awards and the values they see reflected in them. Members have provided input on the award system either in writing or in small gatherings we have organized. These contributions prompted me to think about the ways we can improve the transparency of our processes so that our members can better understand how these awards come to be given. How can we ensure that the award system we have in place uplifts work across the various areas represented in the committee and avoids narrowing the scope of our vision? In partnership with the CEO, Keith Alm; Director of Outreach, Glori Rosenson; Senior Coordinator Member Experience, Liza Boscow; and many of you, we are currently working on a process that we hope will allow us to honor our internationality and capture talent across sectors, i.e., academic, industry, non-profit, foundation, and clinical.

As I think about the ISSCR as whole, a Society comprising nearly 5,000 members, I reflect on how to best serve them across their locations in the world and across various subfields they are interested in and committed to advance. This was particular in our mind as we shaped the 2025 Annual Meeting Program along with the Program Committee and the Chairs Eugenia Piddini and Kathy Cheah. The Chairs were particularly keen that every one of our members finds something stimulating and relevant to them at the annual conference. They sought out international participation and to learn from speakers who may not have had the chance to contribute in the past and whose work spans across the various subfields our members work within. The remarkable work of the entire Committee, Director of Global Events Erika Kowalczyk, and Scientific Program Manager Shuangshuang Du created an annual program in which 70 percent of our invited speakers are first-time ISSCR speakers. Members of our global community are coming together to nourish each other with their inspiring contributions across various fields. These individuals come from 22 different countries, spanning North America, Asia, Europe, Latin America, Middle East, and Africa.

In parallel conversations, we have also brainstormed about current approaches to conferences and where and how we may be missing the voices of our next generations. The chairs of the Early Career Advisory Committee, Alessandro Bertero and Evan Lee Graham, are partnering with the Society of Developmental Biology and the Allen Institute to test a new framework where power reversal, collaborative work, and different formats of talks can gather and inspire the field across generations.

As I look at our future and I relearn what we know, it is not always easy to remember that it is having multiple perspectives present in everything we do that fuels our learning and therefore the heart of our field and our Society. The widening of our reach/membership and participation to make the Society a coalescence of individuals worldwide that, through its activities, inspires and motivates their engagement and advancement of the stem cell and regenerative field. We collectively contribute to the capacity to think, advise, and guide each other across a large number of areas from education to manufacturing, from scientific approach to ethical policy making. These different perspectives and commitments can synergize, but only if they contribute in every phase of the ISSCR’s work. Our Board of Directors and its Executive Committee will benefit from regional and professional diversity. Sessions at our annual meeting that include multiple professional backgrounds and different scientific points of view will advance our field, testifying to the importance of complementary skills and concerns in the work we do. When these sessions also attend to questions that arise from our desire to apply our science while promoting justice and equity, we have the possibility of realizing our full potential as a professional, membership society. We must seek out these complementary areas of expertise in order to tackle important questions in the field. This is the unifying message I want to send.

I hope we never get tired of pausing for self-scrutiny, of slowing down to think about “how” we do things and not just “which” things we do. My recommendations to myself and the field at large is to continue to put pressure on ourselves to seek broader input on initiatives, on nominations to leadership roles, and on our choices about where we host international symposia to name a few. As long as that pressure is on each of us, we will be able to stay in conversation and make decisions that will align our behaviors with our values.

## Acknowledgments

I am deeply grateful to Kym Kilbourne who has been partnering with me through and through the entire year in reflection and expression of the content of these messages. The ISSCR team is one made by colleagues who put their talent and exceptional commitment to the service of us all. In addition to ISSCR team members cited above, I feel deep gratitude to Meredith Albrecht, Chris Barry, Lynnea Brand, Andrew Bremer, Denise de Villa, Dodie Dwyer, Yvonne Fischer, Emilia Gilewicz, Olivia Gomez, Ayesha Khan, Megan Koch, Tyler Lamb, Sarah McNamara, Jack Mosher, Vihar Patel, Andrew Podczerwinski, Kendra Prutton, and Hunter Reed.

